# Agency as a catalyst in pulmonary rehabilitation implementation: Physiotherapists’ perspectives

**DOI:** 10.4102/hsag.v31i0.3262

**Published:** 2026-04-21

**Authors:** Lisa Labuschagne, Alison Lupton-Smith, Pamela Gretschel, Ilse du Plessis

**Affiliations:** 1 Department of Physiotherapy, Groote Schuur Hospital, Western Cape Department of Health and Wellness, Cape Town, South Africa; 2 Division of Physiotherapy, Faculty of Health Sciences, University of Cape Town, Cape Town, South Africa; 3 Division of Physiotherapy, Faculty of Medicine and Health Sciences, Stellenbosch University, Cape Town, South Africa; 4 Division of Occupational Therapy, Faculty of Health Sciences, University of Cape Town, Cape Town, South Africa

**Keywords:** agency, barriers, chronic respiratory disease, facilitators, pulmonary rehabilitation

## Abstract

**Background:**

Chronic respiratory diseases (CRDs) are a major global health burden, and pulmonary rehabilitation (PR) is a well-established, evidence-based intervention that improves functional capacity and quality of life for persons affected. Despite this, access to PR is limited globally and within South Africa. Insight into how PR programmes (PRP) are established and sustained in practice may help explain the persistence of this implementation gap.

**Aim:**

To explore physiotherapists’ experiences of establishing outpatient PRP in the Cape Metropole.

**Setting:**

The study included public and private physiotherapists in the Cape Metropole, Western Cape Province, South Africa.

**Methods:**

An exploratory qualitative descriptive design was employed using semi-structured interviews with six physiotherapists who had attempted to establish outpatient PRP. Data were analysed using reflexive thematic analysis, with Social Cognitive Theory informing interpretation of interactions between environmental conditions, clinician agency and programme outcomes.

**Results:**

Three interrelated themes were identified. Firstly, practice contexts, including funding structures, referral systems, organisational support and physical resources, shaped the feasibility of PRP. Secondly, clinician agency, expressed through motivation, self-efficacy, advocacy and adaptive capacity, influenced how physiotherapists navigated these contextual conditions. Thirdly, PRP followed divergent trajectories over time, with outcomes more predominantly shaped by how individual physiotherapists interpreted early experiences and feedback rather than by context alone.

**Conclusion:**

PRP implementation was shaped by dynamic interactions between contextual conditions and clinician agency. Participants faced similar challenges, however differences in their agency influenced whether PRP were sustained, adapted or remained partial. Strengthening physiotherapists’ self-efficacy, behavioural capability and access to mentorship may support more sustainable PR provision in contexts where services remain limited.

**Contribution:**

This study highlights clinician agency as a critical mechanism in PR implementation and suggests that interventions to expand PR services should address both structural barriers and the development of physiotherapists’ adaptive capacity.

## Introduction

Chronic respiratory diseases (CRDs), such as chronic obstructive pulmonary disease (COPD), are significant causes of mortality and morbidity, carrying high costs to healthcare, especially, in low- and middle-income countries (LMICs) like South Africa (World Health Organization [WHO] 2024). Effective management of CRDs is, therefore, an important priority for the healthcare team to address. Pulmonary rehabilitation (PR) is an evidence-based approach to the management of CRD. Pulmonary rehabilitation assumes an individualised, inter-disciplinary management intervention and comprises of education, exercise and behaviour change interventions and various other therapies (McCarthy et al. 2015; Rochester et al. 2015; Spruit et al. 2013). Pulmonary rehabilitation has been proven to be an effective and essential part of the multifactorial management of chronic respiratory conditions, improving both health-related quality of life (HRQOL) and exercise capacity, and reducing healthcare utilisation costs (McCarthy et al. 2015; Rochester et al. 2015). In many high-income countries, PR is integrated within established referral pathways and delivered through hospital-based, community-based and increasingly home-based or tele-rehabilitation models (Holland et al. 2017; Nici & ZuWallack 2010; Spruit et al. 2013). Despite this, even in well-resourced health systems, referral rates and uptake remain suboptimal, with implementation challenges related to funding, clinician awareness and patient engagement widely reported (Desveaux et al. 2015; Keating, Lee & Holland 2011). In LMICs, these challenges are amplified by resource constraints, limited specialist training, fragmented referral systems and competing health priorities (Bickton & Shannon 2022; Frisk et al. 2022; Malla et al. 2023; Méndez et al. 2023; Milner et al. 2018). Across the African continent, PR remains markedly underdeveloped, with limited published data and sparse programme availability (Bilungula et al. 2022). In South Africa specifically, evidence regarding the establishment, sustainability and contextual adaptation of PR programmes is scarce. While international literature has explored structural barriers to PR implementation, far less is known about how clinicians in resource-constrained contexts navigate these challenges in practice. The implementation and success of PR is influenced by complex interactions between healthcare environments, clinician decision-making and patient engagement (Hug et al. 2024). Approaches that focus solely on structural barriers provide limited insight into why similar conditions can result in different implementation outcomes. Social cognitive theory (SCT) offers a useful lens for understanding such variation, emphasising the reciprocal interaction between environmental conditions, individual beliefs and capabilities and behaviour (Bandura 1986). In this study, SCT informed the interpretation of how practice contexts, clinician agency and programme trajectories interacted over time to shape PR delivery.

Understanding how PR programmes are established and sustained in practice is essential to addressing this implementation gap. This study, therefore, explored the experiences of physiotherapists who had attempted to set up outpatient pulmonary rehabilitation programmes (PRPs) in the Cape Metropole, Cape Town, South Africa.

## Research methods and design

### Design

An exploratory qualitative descriptive (EQD) design (Stebbins 2011) was used to generate an in-depth description of how physiotherapists in the Cape Metropole attempted to establish outpatient PR programmes and how they experienced barriers and facilitators in this process.

### Setting

The Cape Metropole is located in the Western Cape province of South Africa, and includes the City of Cape Town, a number of suburbs, rural areas and small towns. Physiotherapists working in either the public or private sectors in this Metropole were eligible for inclusion if they had provided or attempted to provide adult outpatient PRPs. Physiotherapists who had never attempted PR were excluded.

### Sampling

Participants were initially recruited through convenience sampling, with the aim to invite all physiotherapists in the Cape Metropole to participate. Invitations were sent through professional organisations via the South African Society of Physiotherapy (SASP) Western Cape Branch (including both private and public sectors) and the social media platforms of SASP and Allied Health Workers of South Africa. Additional participants were recruited through snowball sampling. The invitation contained a link to SurveyMonkey, where informed consent was obtained, and screening questions confirmed eligibility for inclusion. All physiotherapists that met the inclusion criteria, and consented, were interviewed. The principles of the Declaration of Helsinki were adhered to at all times (World Medical Association 2024).

### Data generation

Data were generated using individual, semi-structured online interviews using Zoom (Zoom Video Communications, San Jose, California). An interview guide was developed in line with qualitative interviewing guidance described by DeJonckheere and Vaughn (2019), which emphasises the use of open-ended, flexible questioning, iterative probing and the exploration of participants’ lived experiences while allowing space for unanticipated insights to emerge. Interviews were conducted by principal researcher (recorded on Zoom and an additional recording device) between June and August 2021. Offline transcription was completed by the principal researcher ensuring accuracy and familiarity with the data. In order to ensure the trustworthiness of the study, credibility, dependability and confirmability were addressed (Lincoln & Guba 1985). This was done through various means, including a reflective journal documenting non-verbal cues, interview circumstances and personal impressions (thereby enhancing data interpretation by providing valuable contextual understanding), member checking (participants were given opportunity to comment on and clarify the researcher’s interpretation of their information) and peer debriefing of the data and analysis with qualified peer researchers. A meticulous audit trail was kept, and the process of bracketing around the interviews was followed including writing of reflective pieces and deep discussion with research supervisors. Additional contextualisation was provided by the participant screening questionnaire, for example, work setting, professional roles and attempts and experiences in providing PRPs. This assisted in ensuring sufficient scope by aiding prolonged engagement with the data and thick description of the participant’s contexts. Data and backups were stored on access-controlled online platforms, adhering to UCT’s data management policy.

### Data analysis

Data were analysed inductively using thematic analysis (Braun & Clarke 2021). This involved familiarisation with the data, generating initial codes, searching for, reviewing, defining and naming themes and producing the report. Coding and theme development were discussed and refined through consultation with research co-authors. The analysis was informed by SCT as a sensitising framework, guiding attention to interactions between contextual conditions, clinician agency and behaviour.

### Ethical considerations

Ethical approval was obtained from the Faculty of Health Science Human Research Ethics Committee of the University of Cape Town (Ref: 026/2021). Written informed consent was obtained from all participants. To ensure confidentiality, participants were allocated a unique study number, and identifying information was removed from transcripts.

## Results

Eleven physiotherapists completed the screening questionnaire. Of these, three did not consent to the interview, one had not yet attempted PR, and one was outside the Cape Metropole resulting in six participants included in the study. Pseudonyms were allocated to the participants; these and their descriptions are detailed in Table 1. Before presenting the thematic findings, a brief overview of programme characteristics is provided to contextualise the sample. Core programme components were largely consistent across participants, with exercise training being the central element in all programmes. Other programmatic details varied based on the context in which they were delivered, the needs of patients in the programme and the skills and experience of the participants. Despite this, all participants highlighted the importance of a holistic approach to care. Three inter-related themes were identified: Theme One: Practice contexts shaping PR programmes; Theme Two: Agency in the context of PR delivery; and Theme Three: Different outcomes in similar contexts. Together, these themes capture the interplay between contextual conditions, clinician agency and programme outcomes. Theme One describes the organisational and system contexts that shaped what was possible in practice. Theme Two explores the internal drivers and relational supports that enabled or constrained clinicians’ agency. Theme Three demonstrates how these contextual and agentic elements combined in different ways to produce divergent trajectories of success or non-success for PR programmes.

**TABLE 1:** Characteristics of participants.

Participant	Years’ experience	Programme length	Self-description of role and practice	Researcher’s impression	Descriptor
Mandy (Female, 28)	6	January 2019 – current	PR and ICU physiotherapist in private practice, offering a PRP that ‘focused on education, breathing, strengthening and endurance training’. Deemed her programme successful.	Different approach and manner to other participants – not confining herself to the norms but acting freely as she felt appropriate.	Individualised
Kim (Female, 33)	10	May 2020 – current	Owner of a private practice with a special interest in PR. Offering a PRP, but referrals and uptake had not been good. Had made an unsuccessful attempt to provide a PRP previously.	Trying for a second time, fixing previous mistakes and overcoming barriers as she goes.	Optimistic
Sarah (Female, 32)	10	August 2015 – current	A director of a private practice, head of their cardio-PR department and a physiotherapist. Running full-time outpatient PRP ‘incorporating evidence-based assessment and treatment, targeted to the patient’s unique presentation, assisting individuals diagnosed with any chronic respiratory conditions, unexplained breathlessness and undiagnosed breathing pattern disorders, as well as athletes looking to improve performance’. Deemed her programme successful.	Experienced and settled, coming from a place of a successful practice that is well known.	Competence
Ben (Male, 33)	11	2013–2021	Owner of a private practice, providing cardio-PR after cardiac events, cardiac surgery and pulmonary infection as well as in cases of ICU acquired weakness. He did not differentiate cardio-PR from PR. Deemed his programme successful for a time.	Innovative and forward thinking, almost to the point of discussing around the question.	Big picture
Nina (Female, 37)	15	Run during 2018	Owner of a private practice. Her practice provided PRPs that incorporated ‘various types of exercises, while referring to other members of the MDT as needed’. Deemed programme a work in progress, not yet successful.	Resigned. A bit of an ‘it is what it is’ mind-set, pushing through without a fuss despite the challenges.	Stoic
Cindy (Female, 45)	21	February – November 2019	The HOD of a private practice. Not currently offering outpatient cardio-pulmonary services or a PRP but had unsuccessfully attempted to do so previously.	Passion for facilitating something that will bring lasting change, but she thinks she is not the person to run it.	Enthusiastic

PR, pulmonary rehabilitation; ICU, intensive care unit; PRP, pulmonary rehabilitation programme; MDT, multidisciplinary team; HOD, head of department.

### Theme One: Practice contexts shaping pulmonary rehabilitation programmes

Theme One examines how organisational and system-level contexts shaped the feasibility and sustainability of PR programmes. Participants described working within environments that influenced not only whether PR could be offered, but also how much negotiation, risk tolerance and adaptation were required to sustain it. These contextual conditions formed the structural landscape within which decisions about engagement, investment and long-term commitment were made. Across accounts, financial structures were experienced less as absolute barriers and more as ongoing sources of uncertainty. Reimbursement mechanisms were described as inconsistent and unpredictable, requiring negotiation on a case-by-case basis:

‘[*W*]e apply for PMB [*prescribed minimum benefits*] for our patients, and we do explain to them you know sometimes the medical aids will pay, sometimes they don’t.’ (Sarah)

This reimbursement unpredictability shaped how participants assessed the viability of sustaining PR. For some, the perceived return on investment was insufficient relative to the effort required:

‘[*P*]eople put in a lot of effort into it and financially, it’s not viable … the amount of effort [*relative*] to the money that comes in.’ (Cindy)

For practice owners in particular, uncertainty translated into perceived financial risk:

‘[*T*]he potential for crippling financial loss is great and so it, it stops many people from actually investing too much into it.’ (Ben)

Financial feasibility was therefore not only about reimbursement, but about perceived risk and willingness to commit resources in the face of instability.

Referral pathways further shaped programme success and sustainability. Participants described limited awareness of PR among medical practitioners, which constrained patient access and reduced programme momentum:

‘[*W*]e’ve tried to raise awareness among the GP’s [*general practitioners*] but the uptake hasn’t been great especially because we thought those patients that are classified on an MRC scale of less than -, to get them before it really becomes four out of four on an MRC scale, you know get them before they lose that quality of life. We thought the GP’s would be great but the uptake’s been very, very slow so it’s mostly pulmonologists umm specialist physicians in the hospital.’ (Sarah)

Similarly, it was emphasised:

‘[*T*]he main thing is really getting buy-in from the patient’s doctors and getting that referral base that’s been the most difficult challenging part for me.’ (Kim)

And noted the relational implications of limited endorsement:

‘[*I*]f the other doctors aren’t pro PR, it’s very difficult to persuade a patient when it’s not coming from the other health care professionals.’ (Nina)

Referral patterns therefore functioned not merely as a logistical pathway, but as a marker of professional legitimacy. Where endorsement was absent, some participants described needing to assume an advocacy role (as Sarah mentioned), highlighting how hierarchical dynamics within the healthcare system may shape PR uptake. Institutional infrastructure also influenced implementation. The availability and appropriateness of space were interpreted differently depending on practice context. For some, particularly in private practice, capital investment represented a significant structural barrier:

‘[*A*]s a practice owner I would have to invest into a space, where I could have an open area space, it can’t be small, it can’t be enclosed … so there alone is quite a bit of capital outlay.’ (Ben)

In contrast, others reframed resource adequacy through adaptation:

‘[*S*]etting it up on like from a resource point of view didn’t need much, originally you think you need a lot, but then you realise how little you actually do need.’ (Mandy)

These contrasting perspectives suggest that perceptions of what was ‘necessary’ for PR delivery were not fixed, but actively negotiated within specific practice realities.

Organisational culture further shaped whether intentions could be translated into sustained action. Where collegial and managerial support were strong, implementation was described as more feasible:

‘I had the full support of the then directors and being a director now I suppose it hasn’t really changed anything I mean the team has been incredibly supportive from the get go, but I can imagine if you are employed by someone who’s not supportive of what you’re doing the implementation is going to be impossible.’ (Sarah)

At the same time, competing clinical responsibilities introduced additional strain. Balancing inpatient work with programme development required negotiating professional time:

‘[*S*]plitting yourself in two and kinda getting to your hospital work, and pushing this, is something that people do struggle with, and I can see why.’ (Sarah)

‘I’m very busy there [*hospital*] and the numbers at the hospital just go up and down too quickly for me to fit it [*PR*] in.’ (Cindy)

Taken together, these accounts show that PR implementation unfolded within uneven organisational and systemic landscapes. Funding structures, referral cultures, infrastructure, institutional support and time demands simultaneously enabled and constrained what participants could realistically pursue. Rather than functioning as static barriers, these contextual conditions created a dynamic structural environment within which feasibility, legitimacy and sustainability were continuously renegotiated.

### Theme Two: Agency in the context of pulmonary rehabilitation delivery

Theme Two highlights the role of clinician agency in determining how those conditions were interpreted and acted upon. Within similar organisational and systemic environments, participants described markedly different responses to constraint and opportunity. Agency emerged not simply as individual motivation, but as the mechanism through which contextual realities were translated into action.

For several participants, implementation was rooted in a strong internal conviction that PR was necessary. This belief acted as a catalyst, shifting PR from an abstract concept to an actionable commitment:

‘I think if you have a passion for this kind of thing, which I do … that is a big driver you know, that’s something I would say you need.’ (Ben)

Similarly, PR was described as reframing the scope of care:

‘[*N*]ow it’s not just chest physio, now there’s actually hope.’ (Mandy)

These accounts suggest that motivation was rooted not only in professional interest, but in a reimagining of what PR could achieve. Participants frequently linked this commitment to dissatisfaction with existing management approaches. Nina explained:

‘[*T*]he thing is the patients keep coming back with the same issue, and when I read about pulmonary rehab they’re saying there’s no reason to do any more research, we know that it works.’ (Nina)

Echoing this sentiment, a participant described the repeated deterioration of patients as the trigger for change:

‘[*T*]he reason for the implementation of this programme is we kept seeing our patients returning to the hospital sort of looking worse every single time, and you know, kind of thought there must be more we can do for these guys.’ (Sarah)

In these accounts, belief in PR effectiveness functioned as an enabling cognitive driver. However, conviction alone did not automatically translate into implementation. Participants varied in their confidence to operationalise that belief.

For some, self-doubt and perceived clinical risk constrained action. Another participant articulated the emotional dimension of working with severely breathless patients:

‘[*B*]reathlessness can make people so scared and the problem for me in the outpatient setting, if I’m 100% honest, is that I find it scary too! I find exercising breathless people scary.’ (Cindy)

In this instance, fear mediated behaviour. Despite recognising PR’s value, uncertainty around patient safety undermined confidence and limited enactment. In contrast, others described building self-efficacy through experience and reflection. Another highlighted how prior attempts shaped later action:

‘[*T*]hinking about the way that I tried to do it in the previous practice and then moving forward to then opening my own practice.’ (Kim)

Rather than abandoning the idea, earlier setbacks became learning opportunities. Similarly, participants described actively developing behavioural capability through self-directed learning:

‘[*A*]nd that’s when I started researching like what we can do to optimise him … pretty much used him as a bit of a guinea pig.’ (Mandy)

Sarah described a comparable process of benchmarking against international practice:

‘[*S*]o I had a look internationally, saw pulmonary rehabilitation, had a look in South Africa, saw that there was nothing, thought it was terrible, so I thought well we’ve gotta do something.’ (Sarah)

In both cases, agency was enacted through information-seeking and experimentation, illustrating how capability was constructed rather than assumed.

Importantly, participants did not build this capability in isolation. Observational learning and mentorship played a pivotal role in strengthening confidence and expanding perceived feasibility:

‘I got a lot of advice and direction of where to look for information from Sarah.’ (Kim)

Cindy described mentorship as extending beyond advice to practical modelling:

‘[*S*]he was just really good at like allowing us to go watch her programme and sending us information … and how they’ve run their programme, and she gave us lots of support with research articles and she sat with us, she even came to see the venue to check that you know, she thought it was a viable option.’ (Cindy)

Through these interactions, capability was reinforced socially. Agency, therefore, emerged not as an isolated personal trait but as relationally supported practice.

Flexibility, adaptation and perseverance further characterised how agency was enacted over time. Participants described reframing obstacles and revising delivery models in response to contextual realities. A participant reflected on early challenges:

‘[*B*]ut can comfortably say that with hard work, none of them [*initial barriers*] are barriers anymore.’ (Sarah)

This reframing suggests a shift from perceiving barriers as fixed constraints to viewing them as negotiable. Similarly, participants described adapting guideline-based models to local circumstances:

‘[*I*]n our demographic as well, I think we do have to be flexible … in South Africa it’s just not always practical and you’ve gotta customise your programme despite what the guidelines say to suit the person in front of you.’ (Sarah)

Innovation in delivery modes further reflected this adaptive capacity:

‘[*E*]ven now I’m doing a lot of remote pulmonary rehab, and so I’ll do like three or four sessions on Zoom.’ (Mandy)

Across these accounts, agency was expressed through persistence, learning, mentorship, reframing and innovation. It was not static, nor uniformly distributed across participants. Instead, it evolved through interaction with contextual conditions and feedback from practice.

Collectively, these findings suggest that PR implementation was shaped not only by external determinants, but also by how physiotherapists interpreted their environments, mobilised internal and relational resources and recalibrated their actions over time.

### Theme Three: Different outcomes in similar contexts

While Themes One and Two examined context and agency separately, Theme Three integrates these strands to illustrate to illustrate how their interaction produced different programme trajectories over time. Although participants operated within broadly comparable private-sector environments, their PR initiatives evolved in different ways. These differences were not explained by contextual barriers alone, but by how participants interpreted early experiences of uptake, feedback and uncertainty – and how those interpretations shaped subsequent action.

For some participants, early positive feedback functioned as reinforcement. Initial ‘trial and error’ phases did not deter continuation of the programme; rather, visible patient improvement and increasing engagement strengthened commitment. A participant described how early experimentation gradually translated into momentum:

‘[*O*]nce we commenced an inpatient pulmonary rehab programme obviously it was a bit of a trial and error … then I started seeing more and more patients and I just realised if not even just the not just exercise therapy but just also show your patient like what they’re actually capable of besides their respiratory disease. And that’s kind of where, where it took off.’ (Mandy)

Here, reinforcement came not only from programme growth, but also from witnessing patient capability. Improvement became motivational feedback. Similarly, as reflected on the transformation from early uncertainty to sustained fulfilment:

‘[*I*]f I knew then how great it would be or how many people we would be helping at this point, it would have been nice to know then, because there were times where it was very, very quiet, but I can’t really, just that it would become everything that I dreamed that it would.’ (Sarah)

Periods of low visibility or slow uptake were retrospectively reframed as transitional rather than terminal. In these cases, early uncertainty did not undermine agency; instead, small gains assimilated into growing programme success. Learning through trial, reflection and patient response contributed to growth rather than abandonment.

However, similar contextual conditions produced different outcomes for others. Limited referrals, inconsistent funding and reliance on patient self-funding introduced forms of uncertainty that, for some, constrained sustainability. They described the structural tension created by reimbursement systems:

‘I’ve always offered it but it’s not something that was very popular and the big reason is that the medical aids don’t pay, so … patients, they would generally … go … to a step-down facility because then it will be paid for, whereas if they wanted to do pulmonary rehab then it comes from their savings.’ (Nina)

In this instance, the value of PR was not questioned; rather, structural funding mechanisms redirected patients elsewhere. The resulting limited uptake reduced programme momentum.

For some, the issue was less financial than relational and professional alignment:

‘I think that my problem is … I don’t want to run the programme, I want to empower someone else to do it.’ (Cindy)

Here, agency was present but oriented differently. Rather than pursuing programme ownership, she positioned herself as a facilitator for others. This shift in role preference influenced the sustainability and direction of her involvement in PR delivery. Importantly, these contrasting outcomes did not reflect the presence of entirely different barriers. Across participants, funding uncertainty, referral challenges and patient engagement were recurring contextual features. What differed was how these features were interpreted and responded to over time. For some, early reinforcement strengthened self-efficacy and justified further investment. For others, slow uptake or structural misalignment dampened momentum or redirected their efforts. Programme trajectories therefore emerged through an ongoing feedback loop between environment, clinician belief, behaviour and outcome. Early experiences influenced confidence and motivation; these in turn shaped subsequent decisions to persist, adapt, delegate or withdraw. Success and non-success were not fixed states but evolving pathways shaped through iterative interaction.

**FIGURE 1:**
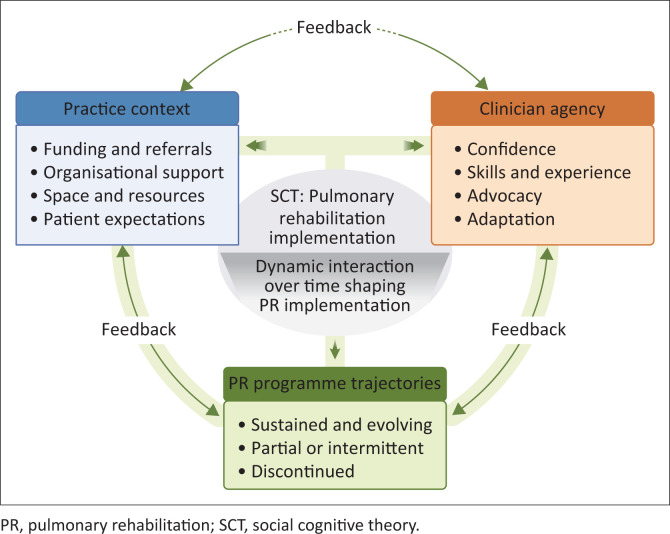
Conceptual synthesis of the study findings illustrating the interaction between practice context, clinician agency and pulmonary rehabilitation programme outcomes through the lens of social cognitive theory.

These patterns underscore that PR implementation cannot be understood as a linear response to contextual barriers. Rather, it unfolds as a dynamic process in which environmental conditions, clinician agency and experiential reinforcement continuously interact over time. These patterns suggest that PR implementation unfolds through a dynamic feedback process in which environmental conditions, clinician agency and reinforcement continually shape one another over time. Figure 1 visually represents this interactional process, illustrating how similar contexts can give rise to divergent programme trajectories.

## Discussion

This study explored how PR programmes are initiated, delivered and sustained within a context where PR remains limited. Drawing on SCT (Bandura 1986), the findings illustrate how PR implementation is shaped by dynamic interactions between environmental conditions, clinician agency and evolving programme outcomes, rather than by any single factor in isolation.

### Environmental conditions as interpreted determinants of implementation

Consistent with SCT, contextual conditions in this study functioned as environmental determinants that shaped participants’ appraisal of feasibility and risk rather than acting as fixed barriers. Funding mechanisms, referral structures, infrastructure and organisational support introduced uncertainty that required ongoing interpretation and negotiation (Jia et al. 2025; Wshah et al. 2024). While international literature consistently identifies these structural factors as constraints to PR access (Desveaux et al. 2015; Milner et al. 2018), the present findings extend this work by demonstrating that structural determinants do not result in uniform effects. Their influence depended on how clinicians evaluated sustainability, financial exposure and professional legitimacy within their specific organisational contexts.

Recent implementation-focused policy statements emphasise that variability in PR uptake is more strongly associated with system integration than programme efficacy (Holland et al. 2021; Rochester 2023). In the present study, referral patterns emerged as particularly influential. Consistent with prior research demonstrating that limited physician awareness limits referral rates, participants described how professional hierarchies shaped both patient access and programme credibility (Keating et al. 2011; Swift et al. 2026). Evidence suggests that structured referral systems and formal embedding within care pathways significantly improve PR sustainability (Hug et al. 2022, 2024; Watson et al. 2023). However, the current findings highlight that even in the absence of formal integration, clinicians may respond in divergent ways – intensifying advocacy or withdrawing effort, reinforcing the interpretive dimension of environmental constraints. In LMICs, structural fragility further compounds these challenges (Bickton & Shannon 2022; Bilungula et al. 2022). The findings support this literature while illustrating how financial insecurity and reliance on specialist referrals amplify perceived implementation risk (Jia et al. 2025; Wshah et al. 2024). Structural conditions therefore shaped not only access, but clinicians’ evaluation of whether PR was viable within their practice realities.

### Agency as enacted and contextually mediated capability

The second major contribution of this study lies in foregrounding clinician agency as an enacted and contextually mediated capacity. Implementation science recognises the importance of ‘local champions’ in sustaining innovations (Desveaux et al. 2015; Greenhalgh et al. 2004), yet often treats agency as a fixed attribute. The present findings, however, demonstrate that agency emerged through iterative processes of experiential learning, mentorship, confidence-building and adaptation, consistent with implementation science frameworks that conceptualise innovation uptake as socially mediated and contextually negotiated (Bandura 2004; Greenhalgh et al. 2004; Jia et al. 2025; May & Finch 2009; Paina & Peters 2012).

Participants who demonstrated stronger self-efficacy and access to experiential learning were more likely to adapt programme models, persist through uncertainty and expand delivery over time. This aligns with Bandura’s (2004) conceptualisation of self-efficacy as a central determinant of sustained behavioural enactment under challenging conditions. Pulmonary rehabilitation research similarly indicates that clinician confidence and exposure to successful delivery models influence referral behaviour and implementation (Milner et al. 2018; Watson et al. 2023). Conversely, fear, role misalignment and perceived clinical or financial risk undermined sustained action (Wshah et al. 2024). Broader innovation research has shown that perceived risk and professional ambiguity significantly reduce adoption likelihood (Greenhalgh et al. 2004; Paina & Peters 2012). The present study extends this understanding by demonstrating how agency interacts reciprocally with environmental uncertainty rather than operating independently of it.

### Implementation as nonlinear and reinforcing trajectories

The third analytic contribution lies in conceptualising PR implementation as emergent and reinforcing trajectories rather than binary success or failure. Early experiences of patient improvement or growing referral visibility strengthened confidence and commitment. Conversely, limited uptake or unresolved uncertainty dampened motivation and redirected effort. Consistent with broader implementation science, health service innovations rarely follow linear trajectories but instead evolve through adaptive, iterative processes shaped by context (Greenhalgh et al. 2004; Paina & Peters 2012). Pulmonary rehabilitation literature similarly emphasises that programme sustainability depends on local integration, system embedding and the capacity to adapt delivery models over time (Holland et al. 2021; Rochester 2023).

Within SCT, this reflects reciprocal determinism: behaviour, environment and personal factors continuously influence one another over time (Bandura 1986, 2004). Importantly, similar contextual conditions produced divergent outcomes depending on how clinicians interpreted and responded to early reinforcement. These observations echo emerging evidence that PR uptake and sustainability evolve through iterative adaptation to context and stakeholder engagement rather than through singular, linear implementation processes (Bilungula et al. 2022; Rochester 2023).

### Implications for pulmonary rehabilitation implementation

These findings suggest that efforts to expand PR services should move beyond addressing structural barriers alone. While improving funding models, referral pathways and training opportunities remains essential, equal attention should be given to strengthening clinician self-efficacy, mentorship and opportunities for experiential and observational learning. Supporting physiotherapists to develop confidence, adaptive capacity and access to mentorship networks may enhance the likelihood that PR programmes are initiated and sustained, even within constrained environments.

### Limitations

This study has several limitations that should be considered when interpreting the findings. The research was conducted within a geographically limited metropolitan area. While physiotherapists from both public and private sectors were invited to participate, only private-sector physiotherapists met the inclusion criteria and consented to participate. This may limit transferability to public-sector or rural settings. The sample was small (*n* = 6) and recruitment relied on convenience sampling, which may have favoured physiotherapists with a particular interest in respiratory care or PR. However, PR services remain scarce within the study context, and the pool of physiotherapists actively involved in PR delivery is inherently limited. The sample therefore reflects the realities of PR availability rather than an attempt at exhaustive representation. The study did not aim to achieve data saturation in the sense of capturing all possible perspectives. Instead, the focus was on developing an in-depth understanding of how PR implementation is shaped by the interaction between contextual conditions and clinician agency. While additional participants may have introduced further variation, the analysis reached a point of conceptual coherence, with recurring patterns across participants that were sufficient to address the study aims. Including physiotherapists who had not considered or attempted PR delivery may have yielded valuable additional insights, particularly regarding non-implementation pathways, and represents an important direction for future research.

### Recommendations

Findings from this study suggest that physiotherapists’ agency plays a critical role in whether PR programmes are initiated and sustained. Physiotherapists working with patients with CRD should be encouraged to consider PR as part of routine care and to apply PR guidelines flexibly in relation to available resources and patient contexts. Access to mentorship from experienced PR physiotherapists may support the development of confidence, behavioural capability and adaptive practice, particularly for those initiating PR in settings where services are limited.

Participants highlighted the importance of advocacy and professional relationships in facilitating PR delivery. Efforts to strengthen collaboration between physiotherapists, medical practitioners and healthcare organisations may improve referral pathways and enhance the perceived legitimacy of PR. Professional bodies, such as the Cardio-Pulmonary Rehabilitation Group of the SASP, could play a coordinating role in fostering mentorship networks and supporting continuing professional development in PR. Engagement with funders and healthcare organisations to explore sustainable models of PR funding may further support programme viability. Future research should explore perspectives of physiotherapists who have not attempted PR delivery, to better understand non-implementation pathways. Additional work examining PR implementation across public-sector and rural contexts, as well as the experiences of other members of the multidisciplinary team, would further inform strategies to expand access to PR services.

## Conclusion

This study demonstrates that the establishment and sustainability of PR programmes are shaped by complex interactions between contextual conditions and physiotherapists’ agency. While participants encountered similar structural and organisational challenges, programme outcomes varied according to how physiotherapists interpreted early experiences, mobilised support and adapted their practice over time. These findings suggest that addressing structural barriers alone is insufficient to expand PR provision. Efforts to strengthen physiotherapists’ self-efficacy, behavioural capability and access to mentorship may enhance the likelihood that PR programmes are initiated and sustained, even within constrained healthcare environments.
